# SARS-CoV-2 variants and the risk of pediatric inflammatory multisystem syndrome temporally associated with SARS-CoV-2 among children in Germany

**DOI:** 10.1007/s15010-022-01908-6

**Published:** 2022-09-01

**Authors:** A. L. Sorg, V. Schönfeld, A. Siedler, M. Hufnagel, M. Doenhardt, N. Diffloth, R. Berner, R. v. Kries, J. Armann

**Affiliations:** 1 Institute of Social Pediatrics and Adolescent Medicine, Division of Pediatric Epidemiology, Ludwig-Maximilians-University Munich, Munich, Germany; 2 University Children’s Hospital, Eberhard Karls University, Tuebingen, Germany; 3 Immunization Unit, Department for Infectious Disease Epidemiology, Robert Koch Institute, Seestr. 10, 13353 Berlin, Germany; 4 Division of Pediatric Infectious Diseases and Rheumatology, Department of Pediatrics and Adolescent Medicine, University Medical Center, Medical Faculty, University of Freiburg, Freiburg, Germany; 5 Department of Pediatrics, University Hospital and Medical Faculty Carl Gustav Carus, Technische Universität Dresden, Dresden, Germany

**Keywords:** PIMS-TS, MIS-C, SARS-CoV-2, Variants, COVID-19, Risk

## Abstract

**Purpose:**

To investigate the relationship between the risk of pediatric inflammatory multisystem syndrome temporally associated with SARS-CoV-2 (PIMS-TS) in children and the predominance of different SARS-CoV-2 variants of concern (VOC) over time.

**Methods:**

In relation to the Alpha, Delta, and Omicron VOC phases of the pandemic, the risk of developing PIMS-TS was calculated by analyzing data for rtPCR-confirmed SARS-CoV-2 infections reported to the German statutory notification system, along with data captured by a separate, national PIMS-TS registry. Both overall infection rates and age group-specific ratios of PIMS-TS during the different pandemic phases were calculated using the Alpha period as the baseline.

**Results:**

The PIMS-TS rate changed significantly over time. When the Alpha VOC was dominant [calendar week (CW) 11 in March–CW 31 in August 2021], the PIMS-TS rate was 6.19 [95% confidence intervals (95% CI) 5.17, 7.20]. When Delta prevailed (CW 32 in August 2021–CW 4 in January 2022), the rate decreased to 1.68 (95% CI 1.49, 1.87). During the Omicron phase (CW 5 in January–CW 16 in April 2022), the rate fell further to 0.89 (95% CI 0.79, 1.00). These changes correspond to a decreased PIMS-TS rate of 73% (rate ratio 0.271, 95% CI 0.222; 0.332) and 86% (rate ratio 0.048, 95% CI 0.037; 0.062), respectively, in comparison to the Alpha period. Rate ratios were nearly identical for all age groups.

**Conclusion:**

The data strongly suggest an association between the risk for PIMS-TS and the prevailing VOC, with highest risk related to Alpha and the lowest to Omicron. Given the uniformity of the decreased risk across age groups, vaccination against SARS-CoV-2 does not appear to have a significant impact on the risk of children developing PIMS-TS.

## Introduction

While children and adolescents usually experience mild courses of Coronavirus disease (COVID-19) and hospitalization rates among this group generally are low [1, 2], pediatric inflammatory multisystem syndrome temporally associated with SARS-CoV-2, (PIMS-TS, also known as MIS-C), has emerged as one of the most important factors affecting morbidity related to SARS-CoV-2 in this age group. From the beginning of the pandemic in early 2020 through May 2021, a period during which the Wuhan and Alpha virus variants dominated, the risk of developing PIMS-TS was 1:4000 infected children and adolescents in Germany [1]. PIMS-TS is a severe complication following SARS-CoV-2 infection. Its main characteristics are fever and multiple organ dysfunction—in particular, cardiac dysfunction and severe shock—due to the patient’s hyperinflammatory state [2]. A high proportion of patients require intensive medical care [3, 4]. Intravenous immunoglobulins (IVIG), steroids, and, in refractory cases, biologicals such as TNF-alpha- or IL-1-blockers are often used as treatment. With these treatments, the majority of patients have good outcomes [5]. Mortality rates are low and patients usually recover within days [6]. Long-term sequelae have been reported in just a minority of cases [7].

Despite higher infection rates with the Delta and especially the Omicron variant of SARS-CoV-2, the number of PIMS-TS does not appear to have increased proportionally [8, 9].

Here we address the question of whether the risk of PIMS-TS may be related to the SARS-CoV-2 strain prevalent in a select geographic region at a certain time. Examining data for the period March 2021-April 2022, a total of 57 calendar weeks, we compared PIMS-TS rates, (calculated from PIMS-TS case registry numbers and based upon reported, laboratory-confirmed SARS-CoV-2 infections), with the different phases of dominant SARS-CoV-2 variants of concern (VOC) in Germany. Given that the vaccination of 12–17 year-olds in Germany began being recommended in August 2021, we also asked whether or not the PIMS-TS rate in this adolescent group decreased more markedly than in younger age groups who did not yet have universal access to SARS-CoV-2 vaccination.

## Materials and methods

For our analysis, we aggregated data from two different sources. From the statutory notification system of laboratory-confirmed SARS-CoV-2 infections managed by the Robert Koch Institute (RKI), Germany's national public health institute [10], as well as from a national registry capturing hospitalized pediatric PIMS-TS cases, one maintained by the German Society for Pediatric Infectious Diseases (DGPI) [11], we examined the numbers of reverse transcription-polymerase chain reaction (rtPCR)-confirmed SARS-CoV-2 infections.

In Germany, it is obligatory to report SARS-CoV-2 infections confirmed by rtPCR and/or culture isolation to local public health authorities. These reports are recorded in a central database managed by the RKI in Berlin.

During the observation period of our analysis, the testing strategy for school children and adolescents remained consistent and ongoing throughout Germany. This included mandatory routine rapid antigen testing (RAT) performed two to three times a week. In some areas, pooled rtPCR-tests also were carried out. Pool tests were widely implemented in kindergartens, but there were differences among testing strategies in kindergartens depending on the federal state they belonged to. Children with a positive RAT were re-tested by means of a rtPCR; in the event of a positive rtPCR, the case was reported to the statutory notification system.

Since nearly the beginning of the pandemic (May 2020), the DGPI’s PIMS-TS registry has captured data on hospitalizations of patients under 17 years of age who meet the WHO case definition of PIMS-TS. All German pediatric hospitals and departments were invited to participate in the voluntary reporting of data. During the study period, a total of 209 pediatric hospitals participated in the registry; this represents approximately 65% of all pediatric departments in Germany. It should be noted, however, that not all departments treat patients with PIMS-TS but instead may refer them to departments that are able to offer a higher level of care. Via a link on the DGPI website (https://dgpi.de/covid-19-survey-der-dgpi/), survey participants were able to access an electronic case report form on a REDCap (Research Electronic Data Capture) platform hosted at Technische Universität Dresden. In addition to the number of cases, the pediatricians in charge provided information on demographic characteristics, symptoms and clinical signs, treatments (including need for intensive care), disease course during hospitalization, and outcome at hospital discharge.

### Outcome of interest and statistical analysis

In order to assess the potential association between developing PIMS-TS and the different virus variants dominant in Germany, three pandemic periods were defined according to calendar weeks (CW): CW 11–31 in 2021 (Alpha VOC); CW 32 in 2021 to CW 4 in 2022 (Delta VOC); and CW 5–16 in 2022 (Omicron VOC) [12]. The dominant variant was defined as the one accounting for > 50% of the infections during the respective CW. In CW 11 in 2021, mandatory, routine SARS-CoV-2 screening via RAT for school children and adolescents was introduced in Germany. This testing continued until CW 16 in 2022. Since PIMS-TS is known to occur on average approximately four weeks following infection, time periods were adjusted accordingly.

In order to identify potential vaccination effects, the outcome of interest was calculated in total and then stratified by age group. Age groups were defined according to the COVID-19 vaccination recommendations for children in Germany. For age-specific analysis, the age groups “under 5 years of age”, “5–11 years of age” and “12–17 years of age” were used. Starting on June 10, 2021, vaccination for children in Germany began being recommended for 12–17-year-olds with pre-existing conditions. On August 19, 2021, this initial guidance was replaced by a general recommendation to vaccinate all adolescents aged 12–17 years old [13]. During the overall period studied, no general vaccination recommendation was in place for children 5–11 years old; only for those in this age group who had pre-existing conditions was vaccination recommended (starting on December 17, 2021). To date, no vaccine has been licensed for use in children under 5 years old in Germany.

By dividing the number of PIMS-TS cases by the number of reported rtPCR-confirmed SARS-CoV-2 infections, the rate of PIMS-TS per 10,000 SARS-Cov-2 infections was calculated with a 95% confidence interval (95% CI). In addition to determining the absolute risk, we calculated the overall and age group-specific rate ratios with 95% CI of PIMS-TS rates during the different observation periods, using as a baseline the initial period when Alpha was the dominant SARS-CoV-2 variant. Also calculated was the respective percentage decrease in risk during each observation period as compared to the initial Alpha period. Lastly, we analyzed rate ratios for the Omicron period with the rate of PIMS-TS in the Delta period as the baseline. These analyses were performed using SAS Version 9.4 (SAS Institute, Cary, NC, USA).

### Ethics approval

All data for these analyses were collected in line with the principles of the Declaration of Helsinki and made available in anonymized form for scientific purposes. The DGPI registry was approved by the Ethics Committee of the Technische Universität (TU) Dresden (BO-EK-110032020) and was assigned clinical trial number DRKS00021506.

## Results

As of December 31, 2020, there were 13,863,259 children and adolescents under the age of 18 living in Germany: 3,975,333 are < 5 years old; 5,375,407 are 5–11 years old; and 4,512,519 are 12–17 years old [14]. During the total observation period, (CW 11 in 2021 through CW 16 in 2022), a total of 5,224,183 SARS-CoV-2 infections were reported in children < 18 years old.

During the same period, 531 pediatric cases (< 18 years old) with PIMS-TS were reported by German pediatric hospitals, indicating an overall average PIMS-TS rate of 1.02 (95% CI 0.93, 1.10) per 10,000 infections. However, the PIMS-TS rate changed significantly over time. When the Alpha VOC was dominant (CW 11 in March CW 31 in August 2021), the PIMS-TS rate was 6.19 (95% CI 5.17, 7.20). When Delta prevailed (CW 32 in August 2021–CW 4 in January 2022), the rate decreased to 1.68 (95% CI 1.49, 1.87). During the Omicron phase (CW 5 in January–CW 16 in April 2022), the rate fell further to 0.89 (95% CI 0.79, 1.00) (Table 1). These changes correspond to a decrease in PIMS-TS rate of 73% (rate ratio 0.271, 95% CI 0.222; 0.332) and 86% (rate ratio 0.048, 95% CI 0.037; 0.062), respectively, as compared to the Alpha period. The rate ratio of pediatric PIMS-TS in the Omicron period as compared to the Delta period was 0.18 (95% CI 0.14; 0.22). Rate ratios were nearly identical for all age groups.

**Table 1:**
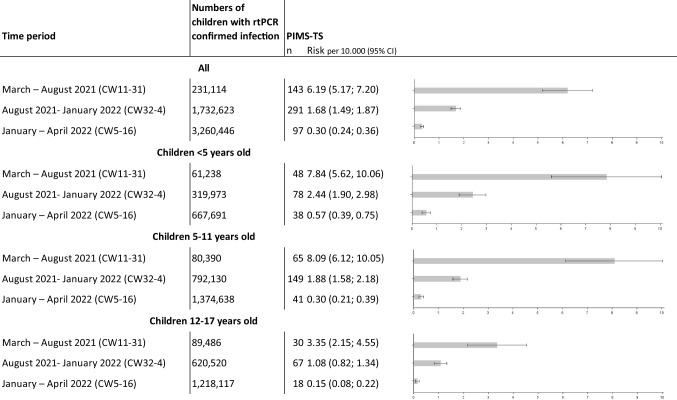
Rate of pediatric PIMS-TS during different periods of the SARS-CoV-2 pandemic with different dominant VOC in Germany, overall and stratified by age groups.

**Fig. 1:**
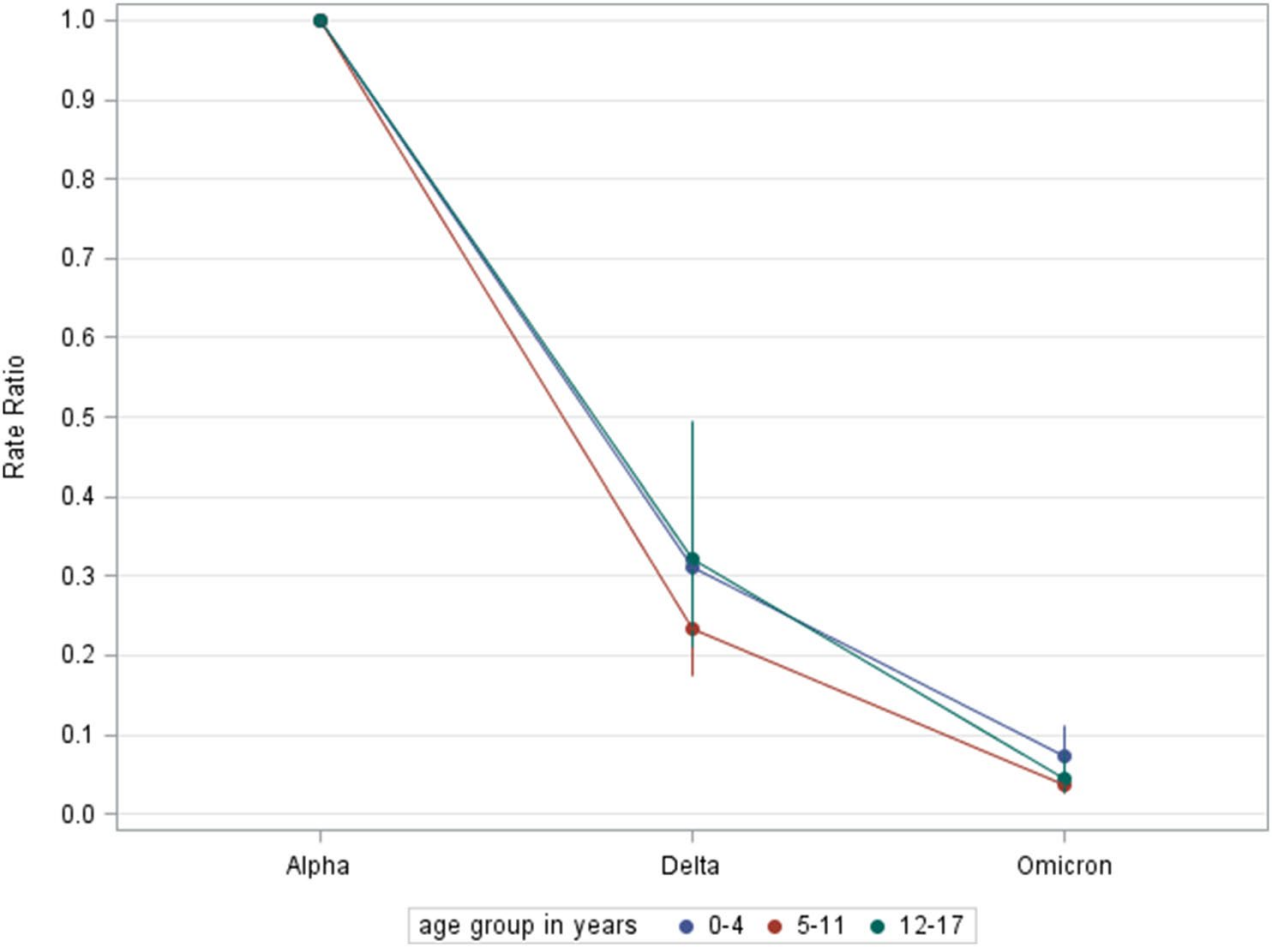
Rate ratios of the pediatric PIMS-TS rate per age group during different periods of the SARS-CoV-2 pandemic with different dominant VOC in Germany: Alpha—calendar week (CW) 11 in March to CW 31 in August 2021; Delta—CW 32 in August 2021 to CW 4 in January 2022; Omicron—CW 5 in January to CW 16 in April 2022

With regard to age group-specific results, absolute risk for PIMS-TS differed. In all phases of the pandemic, absolute risk was greater in the two younger age groups (children < 12 years) than it was in children aged 12–17 years old (Table 1). Over time, however, the decrease in rate ratios was nearly identical for all three age groups (Fig. 1). The age group-specific rate of PIMS-TS decreased in the range of 68% to 77%, with rate ratios of 0.32 (95% CI 0.21; 0.50) to 0.23 (95% CI 0.17; 0.31) during the second period (CW32–4). During the third period (CW5–16) as compared to the initial period (CW 11–31), it went down by 93% to 96% with rate ratios of 0.07 (95% CI 0.05; 0.11) to 0.04 (95% CI 0.03, 0.08).

**Fig. 2:**
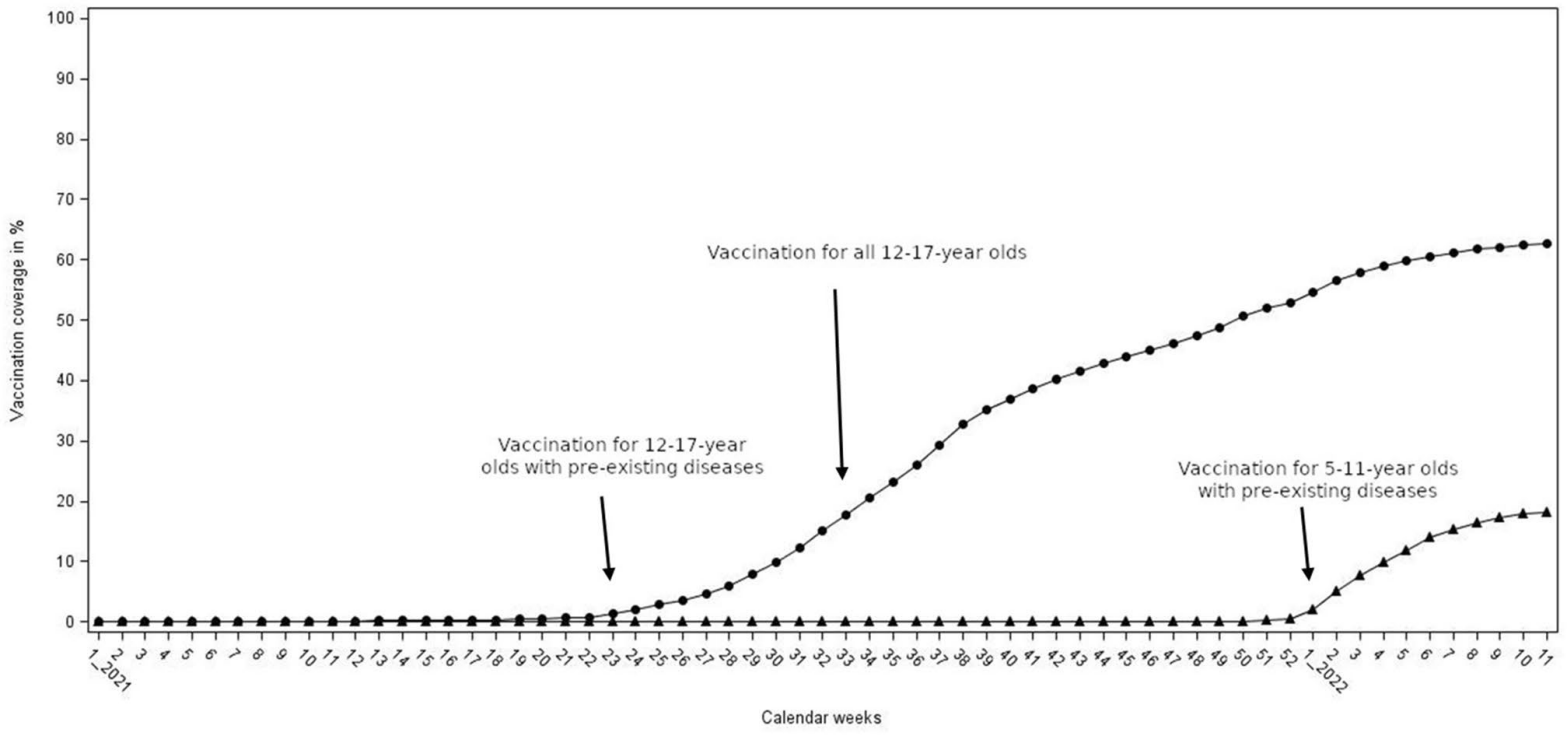
Vaccination coverage for 2 vaccine doses in children < 18 years of age in Germany from 2021 to calendar week (CW) 11 in 2022. Round icons indicate vaccination coverage in 12–17 year-old children and adolescents, while triangular icons indicate vaccination coverage of 5–11 year-olds

Since the introduction of the SARS-CoV-2 vaccination recommendation for all adolescents 12–17 years old in Germany on August 19, 2022, vaccination coverage for basic immunization with two vaccination doses steadily has increased in this age group. By the end of the observation period, coverage had risen to over 60% of all adolescents (see also [15]). Figure 2 shows vaccine coverage for basic immunization, which usually covers two consecutive vaccinations with BNT162b2. The recommendation to vaccinate 5–11 year-old children, issued in early 2022, only applied to children with pre-existing conditions. As a result, the basic immunization rate in this age group only reached approximately 20% by the end of the observation period (Fig. 2).

## Discussion

Our analysis reveals that over the course of the COVID-19 pandemic, the risk of children developing PIMS-TS decreased along with the emergence of each successive variant. At the beginning of the observation period, the rate of PIMS-TS was 6.19 per 100,000 infections; by January 2022, it had decreased to 0.89. During the three observation periods examined, different SARS-CoV-2 variants were in circulation. The data suggest a strong correlation between risk for PIMS-TS and the prevailing VOC, with the highest risk existing in connection to Alpha and the lowest in relation to Omicron. Uniformity in the decrease in PIMS-TS across age groups over time does not, however, indicate that vaccination plays a major role in reducing the risk of infected children developing PIMS-TS. Rather, SARS-CoV-2 vaccination appears neither to increase nor decrease the risk of PIMS-TS. This is consistent with analyses from the UK [16] and Denmark [17], which also found a lower risk of PIMS-TS during the periods when Delta and Omicron were dominant variants.

Because the true number of SARS-CoV-2 infections is both unclear and difficult to determine, the challenge for studies like ours is the common denominator. In both our study and the one from the UK, it was the total number of reported rtPCR confirmed infections used for analysis. This seems sensible as long as the relative rate of PIMS-TS during the Delta and Omicron phases remains the focus. Interestingly, the rate ratios in the UK and Germany so far have been nearly identical.

The Danish study had different strengths than ours. While our study was strictly ecological, the Danish data allowed for a disentanglement of vaccinated and unvaccinated cohorts. Unlike ours, it had limitations in relation to its low participation numbers and its corrections for unidentified cases in the incidence data. Interestingly, however, the Danish study found virtually the same risk for PIMS-TS in the wild virus period, as had been shown previously [1]. Although the numbers were too small for them to calculate rate ratios, a cursory review of their data for the Delta and Omicron periods suggests reductions similar to both our analysis and that from the UK study. Although the Danish study was able to break down the data by age groups, here again the number of cases was too small to provide a conclusive analysis. By contrast, our age group-specific data are based upon substantial higher case numbers and do not suggest there to be a more significant reduction among the vaccinated cohorts.

Despite two-dose-vaccination coverage among 12–17 year-old adolescents having reached over 60% in Germany by April 2022, there was no parallel indication of a reduction in the PIMS-TS rate among this age group. Interestingly, a vaccination rate of over 50% in this age group already had been attained by December 2021—i.e., well before the emergence of Omicron in Germany. Since vaccination reduces the absolute risk of infection, it is possible that vaccination does impact the absolute risk of PIMS-TS, but this is not a factor we were directly able to control for in our data. Given the low and rapidly waning effectiveness of vaccines vis-à-vis preventing mild disease among children infected with Omicron [18, 19], we presume the effect to be low.

The strength of our analysis lies in its use of population-based data and the inclusion of over five million confirmed SARS-CoV-2 infections detected by means of a uniform, widely available, testing strategy during the observation period–with more than 30% of seropositive cases identified as of March 2021 [20]. Since May 2022, routine screening tests no longer have been mandatory in German schools. As a result, in the future analyses such as the one presented here will be far more difficult to carry out with any validity, since the number of unreported cases will change over time. The testing strategy being implemented at any given moment also has an impact. With our study, our hope is that our epidemiological findings will shed light on the pathophysiology of PIMS-TS. Since the SARS-CoV-2 variants mainly differ in their mutations within the viral S-protein, it is possible that the immunological response to the S-protein may be responsible for the hyperinflammatory state displayed with PIMS-TS. This hypothesis is additionally supported by true PIMS-TS following vaccination with the mRNA-vaccine coding for the S-protein occasionally have been observed without prior infection having taken place.

### Limitations

Our study contains some limitations. First, even though a RAT screening strategy for all children in Germany was routine and mandatory during the period of our analysis, a degree of ascertainment bias for the infection rate cannot be completely ruled out. However, given the degree of the variant-effect on the risk of PIMS-TS, it is unlikely to have been caused by variations in rtPCR-tests conducted.

Second, our analysis cannot provide data regarding the absolute risk of PIMS-TS in relation to different SARS-CoV-2 variants, since even with a uniform, routine screening strategy, a degree of underreporting in the numbers of the statutory notification systems remains unavoidable. For this reason, we only are able to describe relative changes. With reductions of approximately 70–90%, however, the impacts demonstrated were substantial. Previous estimates of the absolute risk of PIMS-TS in Germany [1] and the US [16] have shown the absolute risk of the original Wuhan and Alpha variants to be approximately 1:4,000. By contrast, the absolute risk for PIMS-TS related to Omicron infections appears to be between 1:40,000 and 1:80,000.

Additionally, the DGPI’s PIMS-registry is based upon voluntary notification. Although the vast majority of hospitals caring for patients with PIMS-TS have been reporting their cases to this registry, we cannot rule out a level of underreporting. Given that there are no other reporting systems for PIMS-TS cases in Germany, we are unable to validate the reports’ completeness. Nevertheless, even in the event of underreporting, it is important to note that reporting has remained consistent over time; relative trends therefore should not be impacted.

## Conclusion

Our data confirm the results of other ecological studies suggesting that the risk of developing PIMS-TS appears to be reduced in relation to the Delta and Omicron variants. Mutations of the SARS-CoV-2 virus seem to significantly impact the risk for PIMS-TS. The strength of our analysis lies in the denominator of SARS-CoV-2 infection, which allows the assessment of PIMS-TS risk by infection. The effect of COVID-19 vaccination on the risk of developing PIMS-TS seems less pronounced and relevant than the variant effect; this, however, cannot be directly deduced from our data.
